# Graph structure analysis of speech production among second language learners of Spanish and Chinese

**DOI:** 10.3389/fpsyg.2022.940269

**Published:** 2022-09-08

**Authors:** Mona Roxana Botezatu, Janaina Weissheimer, Marina Ribeiro, Taomei Guo, Ingrid Finger, Natalia Bezerra Mota

**Affiliations:** ^1^Department of Speech, Language and Hearing Sciences, University of Missouri, Columbia, MO, United States; ^2^Brain Institute, Federal University of Rio Grande do Norte, Natal, Brazil; ^3^Research Department at Motrix Lab, Motrix, Rio de Janeiro, Brazil; ^4^State Key Laboratory of Cognitive Neuroscience and Learning, Beijing Normal University, Beijing, China; ^5^Department of Modern Languages, Federal University of Rio Grande do Sul, Porto Alegre, Brazil; ^6^Department of Psychiatry and Legal Medicine, Federal University of Rio de Janeiro (UFRJ), Rio de Janeiro, Brazil

**Keywords:** bilingual language production, second language proficiency, graph structure analysis, Spanish, Chinese, English

## Abstract

Language experience shapes the gradual maturation of speech production in both native (L1) and second (L2) languages. Structural aspects like the connectedness of spontaneous narratives reveal this maturation progress in L1 acquisition and, as it does not rely on semantics, it could also reveal structural pattern changes during L2 acquisition. The current study tested whether L2 lexical retrieval associated with vocabulary knowledge could impact the global connectedness of narratives during the initial stages of L2 acquisition. Specifically, the study evaluated the relationship between graph structure (long-range recurrence or connectedness) and L2 learners’ oral production in the L2 and L1. Seventy-nine college-aged students who were native speakers of English and had received classroom instruction in either L2-Spanish or L2-Chinese participated in this study. Three tasks were used: semantic fluency, phonemic fluency and picture description. Measures were operationalized as the number of words per minute in the case of the semantic and phonemic fluency tasks. Graph analysis was carried out for the picture description task using the computational tool *SpeechGraphs* to calculate connectedness. Results revealed significant positive correlations between connectedness in the picture description task and measures of speech production (number of correct responses per minute) in the phonemic and semantic fluency tasks. These correlations were only significant for the participants’ L2- Spanish and Chinese. Results indicate that producing low connectedness narratives in L2 may be a marker of the initial stages of L2 oral development. These findings are consistent with the pattern reported in the early stages of L1 literacy. Future studies should further explore the interactions between graph structure and second language production proficiency, including more advanced stages of L2 learning and considering the role of cognitive abilities in this process.

## Introduction

Much of what is known about speech production comes from the study of single word and sentence production. The production of units of language above a single sentence (i.e., discourse) has received less attention in the literature, even though it represents one of the most complex forms of communication. Speakers engage in the production of spontaneous monologic speech for pragmatic purposes, such as describing a scene or event, giving instructions, telling a story, or arguing for a point of view. A critical part of everyday conversation, monologic speech is a complex task that presents distinct demands on speech planning and production, involving multiple stages of processing. The most pervasive model of speech production (Levelt’s, 1999) “blueprint of the speaker,” and theories of discourse production (e.g., Eggins and Martin, 1997; Halliday, 2004; Sherratt, 2007) agree that the stages of speech production include the selection of a topic/message, the retrieval of relevant information which is then shaped into a logical structure, the selection of the lexical items and grammatical features that map onto the message content, the specification of the phrase structure of each utterance, along with the retrieval of phonological representations of lexical items and the motor execution of the phonetic plan. Interactive effects among these processing stages have been documented, particularly in the speech production literature (for a review, see Goldrick, 2006), suggesting that the distinct stages of processing may influence one another. The current study aims to add to this literature by evaluating how lexico-semantic processes may influence structural aspects of discourse, such as the connectedness of speech produced in continuous sequence. Connected speech may be thought of as the “the rapid, smooth, accurate, lucid and efficient translation of thought or communicative intention into language under the temporal constraints of on-line processing” (Lennon, 2000, p. 26). For the purpose of the current study, we define connected speech as the continuous sequence of spoken words that occurs in monologic discourse.

### Production of connected speech in the second language

The production of connected speech is highly automatized in the native language (L1), yet remains open to the influence of age and education (Le Dorze and Bedard, 1998). In the second language (L2), the production of connected speech is not fully automatized (Kormos, 2006) as a consequence of limited L2 proficiency, best reflected in measures of lexical complexity (Lu, 2012; Kang, 2013; Révész et al., 2016), grammatical complexity (Hahne, 2001; Iwashita, 2006; Rossi et al., 2006; Gan, 2012; De Clercq and Housen, 2017) and phonological encoding (Wong et al., 2021). Critically, the efficiency of L2 lexical access, operationalized as L2 vocabulary knowledge (Hilton, 2008; Koizumi and In’nami, 2013; Uchihara and Saito, 2019) and retrieval speed (De Jong et al., 2013), plays a key role in determining the quality of L2 speech (Kormos, 2006; Liu, 2020). The efficient retrieval of L2 lexical items is dependent not only upon L2 vocabulary knowledge (Hilton, 2008), but also on the ability to resolve high levels of competition from the more dominant L1 (e.g., Meuter and Allport, 1999; Misra et al., 2012), which is co-activated and competes for selection (e.g., Costa et al., 2000; Hoshino and Kroll, 2008; Colomé and Miozzo, 2010). Additionally, speakers have to deal with a limited amount of cognitive resources to provide the system with the necessary energy to operate. This makes L2 speech production even more demanding in the case of beginner L2 speakers, since they have to allocate a great amount of cognitive resources to mobilize lexical, syntactic and phonemic searches while trying to meet the demands of real-time communication (Green, 1986; Green and Abutalebi, 2013). In this sense, we can think of L2 proficiency as a bottleneck that speakers need to reach in order to further be able to employ discourse strategies as a next step in communication.

Emergent findings from research on second language acquisition have revealed a positive relationship between L2 lexical access and various measures of L2 speech quality, such as fluency, accuracy and complexity (Liu, 2020). Yet it is unclear whether this relationship is specific to the weaker, non-dominant L2, or whether it is also encountered in the dominant L1. The current study tested the hypothesis that unlike the native language, where connected speech production is highly automatic, connected speech production in the weaker L2 is highly dependent upon L2 vocabulary knowledge, regardless of the structural distance between speakers’ two languages (e.g., structurally similar languages: English and Spanish; structurally dissimilar languages: English and Chinese). To test this prediction, the current study employed graph structure analysis to investigate the relationship between discourse connectedness and classic measures of lexical diversity (i.e., semantic and phonemic fluency) in speakers’ L1 and L2 in two groups of college-aged L2 learners: native speakers of English who received classroom instruction in either L2-Spanish or L2-Chinese.

### Measures of speech connectedness

**FIGURE 1:**
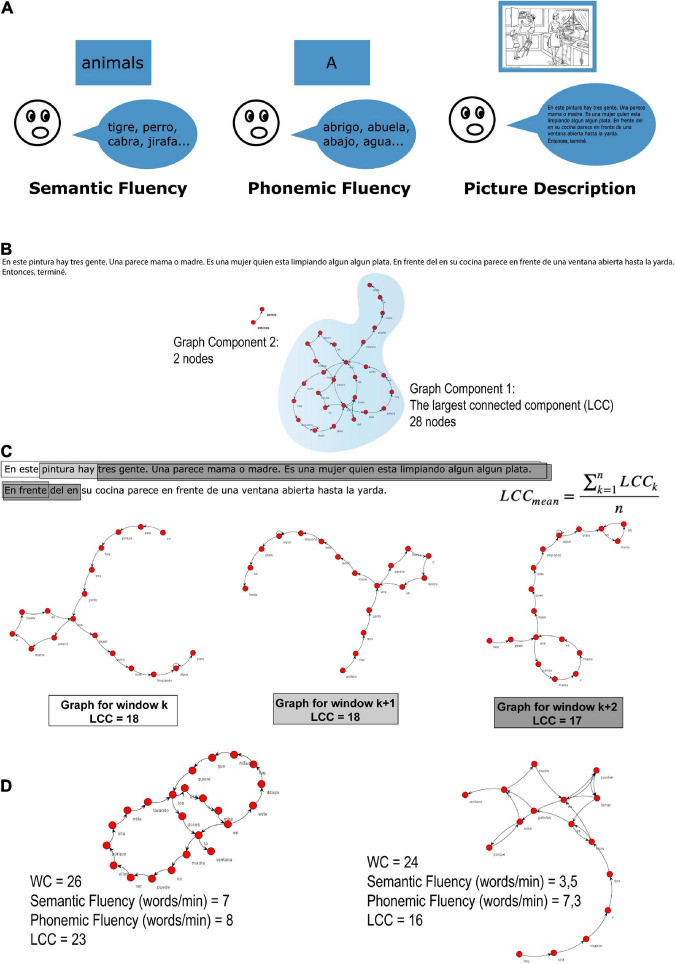
Verbal fluency tasks and graph analysis procedures. **(A)** Semantic fluency, phonemic fluency and picture description were operationalized as the number of words per minute. **(B)** An illustrative example of a graph from a text considering interruptions (here, when there is an interruption from the oral narrative, the following text after the interruption is transcribed in another line). If there are no repeated words, there will be two different components. The LCC counts the number of nodes inside the largest connected component (LCC, indicated by the blue shade). **(C)** To control for verbosity, narratives were analyzed using a moving window of a fixed word length (20 words) with a step of two words. LCC is averaged over the text windows. An example of a text divided into windows of 20 words, jumping two words to the following window. After computing all the 20-word graphs, the average of all the LCCs from all the windows was calculated (as shown in the equation). **(D)** Representative examples of graphs of two bilingual subjects [English (L1) and Spanish (L2)], with different performances in fluency.

To measure discourse connectedness, we have employed graph structure analysis, a method originally created to characterize formal thought disorders in clinical populations (Mota et al., 2012, 2014), but also used with monolingual (Mota et al., 2016, 2018) and bilingual children and adults (Leandro, 2020; Lemke et al., 2021). Formal thought disorders are a set of symptoms identified based on the way a narrative is produced. In this sense, evaluating the spontaneous word trajectory in narrative production mirrors the mental processes involved in the planning and production of discourse. Inspired by the description of formal thought disorders, word graph analysis involves the study of word trajectory by means of representing each word as a node and the spontaneous sequence as directed edges (see Figure 1; Mota et al., 2012, 2014). Representing the narrative as a graph makes it possible to calculate topological aspects (e.g., connectedness) that characterize the word trajectory structure based on the recurrence pattern (Mota et al., 2014). The production of discourse involves a certain degree of word association and repetition. Word graph analysis distinguishes between more or less direct word associations by calculating short and long-range recurrences. Short-range recurrences refer to the repetitions of the same word association (edges that link the same pair of nodes), while long-range recurrences represent the number of nodes inside a connected component (or a set of nodes with at least some connection between them) (Mota et al., 2018). Long-range recurrences provide a measure of global connectedness. Applying this method to characterize thought disorders, we found that the higher the connectedness, the lower the cognitive decline associated with mental illness, demonstrating that word graph connectedness may predict a diagnosis of schizophrenia (Mota et al., 2014, 2017; Palaniyappan et al., 2019; Morgan et al., 2021; Spencer et al., 2021), as well as the cognitive decline associated with dementia (Bertola et al., 2014; Malcorra et al., 2021). Moreover, studying the typical development of discourse patterns, we found that connectedness develops in association with general intelligence (IQ), theory of mind and verbal memory performance, predicting reading acquisition months in advance (Mota et al., 2016, 2020).

In the current study, long-range recurrences, measured by the number of nodes (or different words) inside the largest connected component (LCC), were used as a marker of speech connectedness during a spontaneous speaking task. Although the term “connectedness” is more commonly used in the field of mathematics, where it has emerged, we believe that the closest equivalent in psycholinguistics would be “textual cohesion.” It is assumed that the adjacency between lexical items in a discursive fragment, represented and measured here using graph theory, may be an alternative way to obtain a quantitative measure of text unity; that is, of the relationship between the elements that make up its unity and determine its comprehension. As far as we know, in psycholinguistics, there have been few attempts to find linguistic markers of speech connectivity, one being the measure of syntactic complexity in terms of T-Units (Lemke et al., 2021).

Previous work employing these quantitative measures of speech connectedness has revealed that the production of long-range recurrences changes across lifespan and is associated with L2 proficiency. Mota et al. (2018) described the dynamics of short and long-range recurrence during typical development and their association with formal education, which reveals an interesting pattern of speech connectedness across lifespan. The authors showed that short-range recurrences (e.g., the repetitions of the same word associations) decreased during children’s emerging literacy, but increased with advancing age. Conversely, the ability to produce long-range recurrences in a well-connected narrative increased over school years, and maturation is reached only during high school (Mota et al., 2018), but decreased in older adults in typical aging, as well as in dementia (Malcorra et al., 2021).

### Speech connectedness in bilinguals

In the realm of bilingualism, Lemke et al. (2021) investigated the effects of bilingualism and biliteracy on connectedness and syntactic complexity in the written production of 11-year-old Portuguese-English bilingual children. The authors reported a correlation between graph attributes (i.e., connectedness) and the levels of syntactic complexity in both languages, demonstrating that, as children advance in the development of more complex writing strategies in Portuguese, they progress in their written production in English to the same extent. However, the study conducted by Lemke et al. (2021) did not include oral production tasks, only written ones. The current study addresses this methodological gap by investigating oral production through the analysis of graph attributes. Leandro (2020) was the first to extend this line of work to oral production in adult bilinguals and to show an association between measures of L2 oral proficiency and graph attributes in the case of Portuguese-English adult bilinguals. In his study, graph analysis (i.e., long-range connectedness and short-range repetitions) successfully predicted fluency in the continuum between pre-intermediate and near-native levels of L2 speech proficiency. In general, the more fluent speakers were, in terms of number of words per minute, the more connected their speech was found to be and the fewer short-range repetitions the participants produced. However, the author did not evaluate this relationship in the speakers’ L1, Portuguese. Therefore, the present study fills this gap by looking at the interaction between verbal fluency and speech connectedness in both bilinguals’ first and second languages. Additionally, the studies evaluating speech connectedness in bilinguals have solely focused on speakers of structurally similar languages, such as English and Portuguese. The present study extends the analysis to bilingual speakers of structurally similar languages (i.e., English and Spanish) and structurally dissimilar languages (i.e., English and Chinese) to provide a better representation of possible language pairings in emergent bilinguals and to evaluate whether the relationship between connected speech production and lexical retrieval changes as a function of the structural distance between bilinguals’ two languages.

### The current study

The current study tested the hypothesis that connected speech production in the weaker L2 is highly dependent upon L2 lexical retrieval (vocabulary knowledge), regardless of the structural distance between learners’ two languages. Critically, we predicted that the same association would not be found in the L1 because lexical retrieval is highly automatic in the L1 in adulthood. Alternatively, if lexical retrieval is equally challenging in speakers’ L1 and L2, then we should see an association between measures of lexical retrieval and discourse connectedness in both languages.

## Materials and methods

### Participants

A total of seventy-nine college-aged students who were native speakers of English and reported past or current enrollment in L2-Spanish (*n* = 54, mean age = 20.35, SD ± 2.47, 15 male, average age of initial L2-Spanish exposure = 11.06, SD ± 4.22) or L2-Chinese (*n* = 25, mean age = 21.68, SD ± 3.01, 9 male, average age of initial L2-Chinese exposure = 17.4, SD ± 3.04) courses were recruited from the University of Missouri and Beijing Normal University and completed the study for payment. Participants reported normal hearing, normal or corrected-to-normal vision and no history of neurological, language or learning deficits.

### Materials

Speech production was assessed using a picture description task and lexical retrieval was measured using two distinct verbal fluency tasks (i.e., semantic fluency and phonemic fluency), which are described in detail below (see Figure 1A). All participants completed the tasks in English and in the foreign language in which they had received instruction, Spanish or Chinese, respectively. In addition to the discourse and lexical retrieval measures, participants also completed a language history questionnaire (Marian et al., 2007). All materials are presented below. Additional details on the materials and procedures can be found in Botezatu et al. (2022).

#### Picture description

The Cookie Theft scene from the Boston Diagnostic Aphasia Examination (Goodglass and Kaplan, 1983) was used in the picture description task. Participants were given 5 min to produce a narrative describing the picture and were instructed to speak for the entire time. Each trial began with a 1,000 ms blank screen, which was followed by a picture that cued participants to speak for 5 min and ended with a 1,000 ms blank screen. The resulting oral language samples were then transcribed offline by independent raters and scored in terms of average words-per-minute (96% interrater reliability), following the rules for counting words proposed by Nicholas and Brookshire (1993), providing a measure of discourse fluency.

#### Semantic fluency

A minute-long semantic category fluency task assessed retrieval of lexical items. Data samples were transcribed offline by independent raters (98% interrater reliability) and scored separately in participants’ L1 and L2 in terms of the average number of correct responses (excluding simple and inflected repetitions) produced across four named semantic categories (i.e., *animals*, *clothing, fruit, furniture*).

#### Phonemic fluency

A minute-long letter fluency task was also used to measure retrieval of lexical items. Data samples were transcribed offline by independent raters (97% interrater reliability) and scored separately in participants’ L1 and L2 in terms of the average number of correct responses (excluding repetitions and proper names) produced by participants across three named letters (*F*, *A*, or *S* in English; *P*, *M*, or *R* in Spanish; not assessed in Chinese due to no agreed-upon equivalent measure, but imputed using the Multivariate Imputation by Chained Equations R package (Van Buuren and Groothuis-Oudshoorn, 2010).

#### Language history background

Language experience was measured using the Language Experience and Proficiency Questionnaire (Marian et al., 2007). Participants self-rated their L1 and L2 proficiency, learning experience, frequency and context of exposure and use on a scale from 0 (*no proficiency, never*) to 10 (*native-like proficiency, always*). The questionnaire was administered at the end of the testing session, after participants completed all other tasks.

### Data collection procedure

During one in-person testing session, participants completed the picture description, semantic fluency and phonemic fluency tasks in both the L1 and the L2, as well as a language history questionnaire administered in the L1. Participants were tested in the L1 first and L2 second to avoid L1-inhibition following performance in the weaker L2 (Misra et al., 2012). The experimental tasks were presented electronically using the E-Prime 2.0 software (Psychology Software Tools Incorporated, 2012). The Language History Questionnaire was administered electronically using Qualtrics (2019, Qualtrics, Provo, UT).

### Data analysis

#### Proficiency analyses

To characterize the language proficiency and dominance of the participant sample, we compared L2 and L1 proficiency scores for both L2-Spanish and L2-Chinese groups using Wilcoxon Ranksum Tests. The Wilcoxon Ranksum Test is a non-parametric statistical analysis aiming to check the null hypothesis that two independent samples are equal.

#### Graph analyses

The oral narrative transcriptions from the picture description task, which included all the words spoken spontaneously by participants, were coded as a word-trajectory graph using the *SpeechGraphs* software.^1^ The software represents each word as a node and the sequence of words as directed edges (see Figure 1). This computational tool is used to map the spontaneous relationship between different words in a narrative. The method represents a narrative as a graph, allowing for topological characterization. It provides a number of useful measures (i.e., graph attributes), from elementary measures such as the total number of nodes and edges, to connectedness measures, such as the LCC. In the word graph trajectory, the LCC is defined as the largest set of nodes directly or indirectly linked by some path (see Figure 1). The number of nodes (i.e., different words) found in the LCC provides a measure of global connectedness that may be used to evaluate the lexical diversity of a narrative.

As there was no maximum limit for oral reports, to control for word count differences (i.e., verbosity), we analyzed graphs of 20 words, using a step of two words (corresponding to an overlap of 90% between consecutive graphs) to plot the next graph (see Figure 1C). We used a sliding window technique, in which we chose an initial set of 20 words, plotted a graph, moved two words to the next window and plotted the next graph with the following set of 20 words, and so on consecutively, until the complete set of 20 words in the text was graphed. This allowed us to screen the entire text in 20-word consecutive graphs. We then calculated the LCC of all 20 word-graphs and averaged all LCCs from the same reports. Representative examples of graphs of two bilingual subjects [English (L1) and Spanish (L2)], with different performances in fluency were represented in Figure 1D.

#### Statistical analyses

The analysis revealed that the data were not normally distributed (Shapiro-Wilk test). Therefore, Spearman non-parametric analyses were conducted to assess the association between the graph scores (LCC) originated in the Cookie Theft analysis and both semantic and phonemic verbal fluency measures. We corrected the significance level by using the Bonferroni test for 4 comparisons (α = 0.0125). All the analyses were performed in Python 3.9.7 (Van Rossum and Drake, 1995).

## Results and discussion

### Language proficiency

Participants varied on measures of L1 and L2 production (i.e., discourse fluency; semantic and phonemic fluency) and self-reported proficiency ratings on a 10-point scale (see Table 1). Self-reported proficiency ratings revealed that both L2-Spanish and L2-Chinese learners had a relatively low level of L2 proficiency, with a mean score of 3.9 (SD ± 2.62) in the case of L2-Spanish learners, and a mean score of 3.8 (SD ± 2.46) in the case of L2-Chinese learners. The difference between participants’ L1 and L2 fluency scores was evaluated as an additional measure of proficiency. In L2-Spanish learners, Wilcoxon Ranksum Tests revealed a mean difference of W = –8.35, *p* = 7E-17 in the case of L2 Spanish–L1 English phonemic fluency; and of W = –8.93, *p* = 4E-19 in the case of L2 Spanish–L1 English semantic fluency. In the case of L2-Chinese learners, a mean difference of W = –5.85, *p* = 5E-09 in the case of L2 Chinese–L1 English phonemic fluency; and of W = –5.84, *p* = 5E-09 in the case of L2 Chinese–L1 English semantic fluency were found. Taken together, differences in self-reported proficiency ratings and fluency means between the two languages have led us to characterize the present sample as two groups of beginner L2 learners who maintained dominance of their native language, English.

**TABLE 1:** Mean (SD) psycholinguistic data.

Measure	Spanish learners		Chinese learners	
		
	English proficiency	Spanish proficiency	English proficiency	Chinese proficiency
Discourse fluency	157.43 (63.00)	57.33 (27.00)	121.17 (28.37)	77.19 (35.88)
Semantic fluency	18.95 (3.79)	6.21 (2.47)	18.45 (3.89)	7.39 (3.64)
Phonemic fluency	15.81 (3.66)	7.89 (2.48)	16.96 (3.63)	8.57 (3.05)
Average proficiency self-rating (/10)	10 (0)	3.9 (2.62)	10 (0)	3.8 (2.46)

### Speech connectedness

**FIGURE 2:**
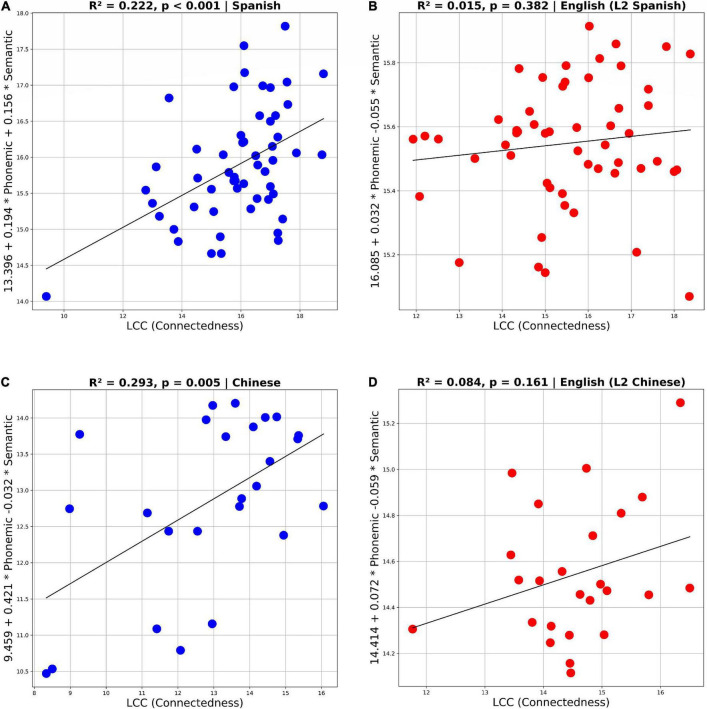
Multiple regression scatterplots showing the contribution of phonemic and semantic fluency to explain connectedness (LCC) in L2 Spanish and Chinese **(A,C)** and in L1 English **(B,D)**.

Multiple regression analysis results in Figure 2 indicate that semantic and phonemic fluency predict speech connectedness only in the case of the L2. Although both semantic and phonemic fluency in L2-Spanish and L2-Chinese significantly contributed to explain speech connectedness in the picture description task (*R*^2^ = 0.222, *p* < 0.001 for Spanish and *R*^2^ = 0.293, *p* < 0.005 for Chinese), in the case of L1-English we see a different pattern, with phonemic fluency and semantic fluency not contributing to the prediction model (*R*^2^ = 0.015, *p* = 0.382 for Spanish/English and R^2^ = 0.084, *p* = 0.161 for Chinese/English). In other words, phonemic and semantic fluency explained 22% of connectedness variance in the spontaneous narratives in Spanish, and 29% of connectedness variance in the spontaneous narratives in Chinese. These results confirm our hypothesis that the speech production of beginner L2 learners is highly dependent on L2 lexical and phonemic retrieval and that connectedness is better explained by fluency in L2 than in L1, regardless of the structural distance between learners’ two languages.

**FIGURE 3:**
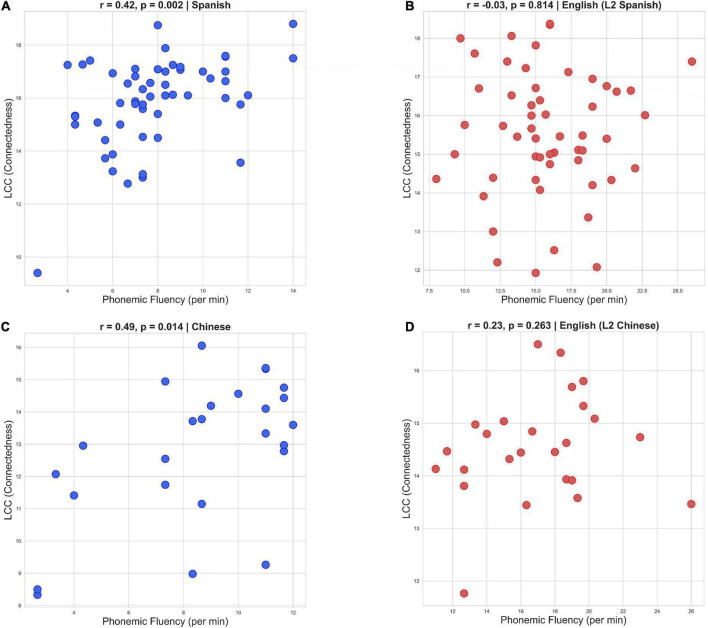
Correlation scatterplots of the Largest Connected Component (LCC) measure in English and L1 Phonemic Fluency **(B,D)** and the LCC measure in the L2 and L2 phonemic fluency (Spanish and Chinese; **A,C**).

The regression analysis results also showed that phonemic fluency was more closely related to L2 connectedness than semantic fluency, especially for Chinese (Coefficient for phonemic fluency = 0.194 and coefficient for semantic fluency = 0.156, in Spanish; and Coefficient for phonemic fluency = 0.421 and coefficient for semantic fluency = 0.032 in Chinese). That led us to run Spearman correlations to evaluate more closely the relationship between phonemic fluency and connectedness in L2 and L1. Again, results revealed positive correlations between long-range recurrences (LCC), measured in the picture description task, and phonemic fluency (R = 0.42, *p* = 0.02 for Spanish and R = 0.49, *p* = 0.014 for Chinese). Once more, these correlations were only significant for the participants’ L2–Spanish and Chinese (see Figure 3), reinforcing the claim we have put forward here that the speech production of beginner L2 learners is highly dependent on L2 lexical retrieval. The fact that L2 connectedness is better explained by phonetic fluency (rather than semantic fluency), regardless of learners’ L2, seems to indicate that L2 learners in the current study relied more on phonetic cues to access lexical structures in order to meet the demands of the picture description task. This finding is consistent with previous reports of a progression from reliance on word form in beginner L2 learners to reliance on word meaning in more advanced L2 learners (e.g., Talamas et al., 1999). Additionally, the results of the current study demonstrate that the relationship between connected speech production in the L2 and L2 lexical retrieval in emergent bilinguals does not change as a function of the structural distance between bilinguals’ two languages.

Taken together, our findings indicate that producing a lower number of long-range recurrences may be a marker of individual differences in the initial stages of L2 oral development, when the ability to produce a well-connected narrative tends to be dependent on a lexical repertoire, which is still under development, in order to incrementally aid connectedness in speech. These findings are consistent with the pattern reported in the early stages of L1 literacy, where the increase in longer recurrences has also been associated with the development of literacy (Mota et al., 2016, 2018). In other words, connectedness in an adult’s L1 speech seems to be well-structured and, therefore, less likely to be explained by variability in the individuals’ L1 mental lexicon. The picture is different in the case of the developing L2, in which variability in individuals’ ability to produce a narrative is linked to vocabulary size (e.g., Hilton, 2008; Koizumi and In’nami, 2013; Uchihara and Saito, 2019) and the speed at which learners can access their lexical repertoire (e.g., De Jong et al., 2013), therefore closely dependent on L2 proficiency (e.g., Kormos, 2006).

The developmental perspective adopted here reveals different strategies to produce a well-connected narrative in a new language. As we can see here, in the initial stages of second language acquisition, phonemic cues seem to play an important role in a naturalistic task such as narrating a scene as a monologue. At more advanced stages we could find different results, as we have presented evidence that the L1 narrative production is not associated with vocabulary retrieval. Also, differences in the bilingual experience or learning context may also reveal other strategies to be differently recruited.

### Limitations and future directions

There are a number of limitations that we would like to acknowledge. First, the design of the current study, which tested participants in the L1 first and L2 second to avoid L1 inhibition following L2 retrieval, likely led to practice effects in the L2. These practice effects may have resulted in increased speech connectedness in the second language, but we cannot test this empirically based on the available data. Future studies should test the influence of practice on speech connectedness in the weaker second language. Second, we had access to a small sample of participants (particularly for the Chinese group), so the results should be replicated with larger samples. Third, we did not have access to participants with higher levels of L2 proficiency, which could reveal differences in the association between narrative production mechanisms and lexical retrieval. More studies with larger and more diverse samples in terms of proficiency levels are needed to advance our current understanding of the association between vocabulary acquisition and naturalistic use of a second language in the production of narratives. Future studies should further explore the interactions between graph structure and second language production proficiency, including more advanced stages of L2 learning and considering the role of cognitive abilities in this process. Associations between cognitive abilities (IQ, memory and theory of mind), academic achievement and speech connectedness have been documented in the past (Mota et al., 2016), revealing that children with higher cognitive and academic scores produced more long-range connections and fewer repetitions. Future research should test these associations in the L2.

## Conclusion

Given that individual difference factors can reveal disparities in L2 speech production among learners, such factors have attracted researchers’ growing interest. Here, we addressed individual differences in L2 speech production by employing graph structure analysis to evaluate the relationship between L2 lexical retrieval and the global connectedness of narratives during the initial stages of L2 acquisition and whether results can be replicated in the dominant L1. The current study contributes to the literature on second language acquisition by demonstrating that in the initial stages of L2 oral development, the connectedness of L2 speech is explained by variability in L2 lexical access. The study also demonstrates that a non-semantic graph strategy may be used to measure dynamics of narrative production in naturalistic settings, promoting the use of computational approaches to track L2 development, allowing for individualized feedback and helping to adjust speech trajectory over time. In addition, speech graphs may offer an alternative to refine the evaluation of L2 speech performance, with teachers and examiners being able to provide a faster and visually informative representation and assessment of learners’ L2 speech production.

## Data availability statement

The raw data supporting the conclusions of this article will be made available by the authors, without undue reservation.

## Ethics statement

The studies involving human participants were reviewed and approved by the University of Missouri Institutional Review Board. The patients/participants provided their written informed consent to participate in this study.

## Author contributions

MB contributed to the data collection, data analysis, and manuscript writing. JW, MR, IF, and NM contributed to the data analysis and manuscript writing. TG contributed to the data collection. All authors contributed to the article and approved the submitted version.

## Footnotes

1 The SpeechGraphs software was created by Mota et al. (2014), originally to be used with psychiatric populations, and is freely available at the following website (https://neuro.ufrn.br/softwares/speechgraphs). The software, which uses plain text as input and generates graphs and mathematical attributes as output, can be used in different platforms, such as Linux, Windows or OSX.
